# The Role of Clinical Pharmacogenetics and Opioid Interactions in Pain Management: Current Evidence and Future Perspectives

**DOI:** 10.3390/pharmaceutics18010059

**Published:** 2026-01-01

**Authors:** Clelia Di Salvo, Giulia Valdiserra, Stefano Balestrieri, Giuditta Beucci, Giulia Paciulli, Giovanna Irene Luculli, Alessandro De Vita, Matteo Fornai, Antonello Di Paolo, Luca Antonioli

**Affiliations:** 1 Unit of Pharmacology and Pharmacovigilance, Department of Clinical and Experimental Medicine, University of Pisa, 56126 Pisa, Italy; clelia.disalvo@phd.unipi.it (C.D.S.); matteo.fornai@unipi.it (M.F.);; 2 Unit of Pharmacology and Pharmacogenetics, Department of Clinical and Experimental Medicine, University of Pisa, 56126 Pisa, Italy; s.balestrieri@studenti.unipi.it (S.B.); g.beucci@studenti.unipi.it (G.B.);; 3 Centro Regionale di Farmacovigilanza e Dispositivovigilanza, USL Toscana Centro, 50122 Firenze, Italy; g.paciulli@studenti.unipi.it; 4 Preclinic and Osteoncology Unit, Bioscience Laboratory, IRCCS Istituto Romagnolo per lo Studio dei Tumori (IRST) “Dino Amadori”, 47014 Meldola, Italy

**Keywords:** opioids, pharmacogenetics, polymorphisms, drug–drug interactions, *CYP2D6*

## Abstract

**Introduction**: Opioids are the most commonly used analgesic drugs for acute and chronic severe pain and are metabolized in the liver via cytochrome P450 (CYP) enzymes and UDP-glucuronosyltransferases (UGTs). **Methods**: A narrative review of the literature was conducted by searching the PubMed database up to December 2025, with English as the only language restriction. Relevant studies were identified using the keywords “opioids,” “pharmacogenetic,” “cytochrome mutations,” and “interactions.” **Results**: Polymorphisms in *CYP2D6* and *CYP3A4* genes can affect the pharmacokinetics, clinical effect, and safety of opioids. Furthermore, enzyme induction and inhibition by concomitant drugs or compounds (herbal products or food) are sources of variability factors in drug response that may be predictable. **Conclusions**: This review article summarizes current evidence on the role of pharmacogenetics and opioid-related interactions, offering a framework to better understand interindividual variability in opioid response and to inform future multimodal approaches.

## 1. Introduction

Opioids are the most used analgesic drugs for acute and chronic severe pain in cancer and non-cancer patients, especially in the elderly suffering from pain-related functional impairment [1,2]. Opioids use increased markedly in 1995 when the U.S. Food and Drug Administration FDA approved oxycodone for the treatment of chronic pain in non-cancer patients. Recent dispensing data show a sustained decline in opioid prescribing in US, with the overall national opioid dispensing rate decreasing from 46.8 prescriptions per 100 persons in 2019 to 37.5 per 100 persons in 2023, although marked geographic variability persists [3]. In parallel, contemporary clinical practice has moved toward patient-centered prescribing and careful risk–benefit reassessment, as emphasized by the Centers for Disease Control and Prevention Clinical Practice Guideline for Prescribing Opioids for Pain (2022) [4].

Opioids induce their analgesic effect by stimulating G protein-coupled receptors (GPCRs), particularly the µ-opioid receptor subtype. Receptor binding modulates neuronal ion channel activity, by altering membrane permeability to K^+^ and Ca^2+^ ions and inhibiting cyclic AMP signaling, thus resulting in an inhibitory effect within both the central and peripheral nervous system and ultimately leading to analgesia [5]. Opioids are also characterized by a narrow therapeutic index, which contributes to a high risk of toxicity [2]. The most frequent adverse effects (AEs) are constipation, nausea, and vomiting, whereas respiratory depression is the most serious AE, although it occurs only at higher doses. Hypotension, vasodilation, bradycardia, and/or QTc interval prolongation are long-term cardiovascular AEs, while further AEs include fatigue, anxiety and depression, osteoporosis, and endocrine disorders [6]. Genes encoding enzymes involved in opioid metabolism, including cytochrome P450 (CYP) and UDP-glucuronosyltransferase (UGT), may harbor several polymorphisms that could affect the opioid metabolic phenotype. Therefore, pharmacogenetics may be fundamental to understanding how allelic variations can influence drug response [7]. In addition, drug–drug interactions (DDIs) are potentially responsible for AEs and can also influence the efficacy of opioids by altering the generation of active/inactive metabolites [2]. In particular, pharmacokinetic interactions deserve attention since opioids are eliminated or bioactivated through hepatic metabolism [7]. This review article provides an overview of pharmacogenetics and opioid interactions that may be useful to better understand interindividual variability in opioid response and to inform future multimodal approaches aimed at optimizing treatment in clinical practice in.

## 2. Materials and Methods

This work is a narrative review aiming to summarize and critically discuss the current evidence on the impact of pharmacogenetics and drug–drug interactions on opioid pharmacokinetics (PK) and pharmacodynamics (PD). For this purpose, a literature search was conducted in PubMed databases up to December 2025, using the English as the only language restriction. The search was conducted combining the Medical Subject Heading (MeSH) and free-text terms related to the topic including “opioids”, “pharmacogenetic”, “cytochrome mutation” and “interactions”. Additional relevant studies not captured in our initial literature search were identified by examining the reference lists of selected papers. We screened review articles, meta-analyses, and original research articles. As this is a narrative review, the study was not registered in PROSPERO, and no formal quality assessment or meta-analysis was performed, in accordance with the recommendations for non-systematic reviews. To ensure the review reflects the most current evidence, we prioritized studies published from 2020 onward while retaining seminal earlier work relevant to mechanisms, PK/PD, and clinically meaningful drug–drug interactions.

## 3. Results

### 3.1. Opioid Pharmacology at a Glance

The term opioids refer to all compounds with the ability to bind to opioid receptors. This category includes natural alkaloids (i.e., morphine and codeine) from the opium poppy, semi-synthetic drugs obtained from natural precursors (e.g., heroin from morphine, oxycodone from thebaine) as well as fully synthetic opioids (i.e., methadone and fentanyl). Morphine, oxycodone, hydromorphone, fentanyl and codeine are primarily used for the management of chronic pain, whereas methadone and buprenorphine are more frequently employed in specific clinical settings, such as treatment of opioid use disorder [8].

In this section, we summarize and critically discuss the current knowledge on opioid pharmacology, with particular emphasis on opioid receptors, metabolic pathways, and efflux transporters involved in opioid disposition and therapeutic response.

#### 3.1.1. Opioid Receptors

Opioid GPCRs are classified into three different classes: µ (e.g., morphine), δ (named after the mouse vas deferens, where it was first identified), and κ (named after ketocyclazocine) [9]. The nociceptin/orphanin FQ receptor (NOP receptor; formerly opioid receptor–like 1, ORL-1) is an additional receptor subtype which is phylogenetically related to the others. Besides nociceptin/orphanin FQ, the NOP receptor binds orphanin FQ, a neuropeptide that activates an opioid-like GPCR. The NOP-N/OFQ system is important in physiological processes due to its wide distribution in the brain, spinal cord, and peripheral tissues [10]. Furthermore, some opioids, such as tramadol and methadone, have additional nonopioid-sites of action [5].

Opioids receptors belong to the class A (rhodopsin-like) GPCR family and are characterized by an extracellular N-terminal domain, seven transmembrane α-helical domains connected by three extracellular and three intracellular loops, and an intracellular C-terminal tail [11]. The main endogenous analgesic ligands are endorphins, enkephalins, and dynorphins [12]. Enkephalins are derived from pro-enkephalin and preferentially act as δ-opioid receptor ligands, whereas endorphins originate from pro-opiomelanocortin and primarily bind to the μ-opioid receptor (MOR). In contrast, dynorphins are derived from prodynorphin and display high selectivity for the κ-opioid receptor subtype. All opioid receptors modulate pain by inhibiting voltage-gated Ca^2+^ channels and/or activating K^+^ channels, resulting in the inhibition of neuronal excitability [13]. Upon activation of opioid receptors, coordinated phosphorylation of the receptor by specific GPCR kinases occurs. After the interaction of the phosphorylated receptors with *β*-arrestin 1 and *β*-arrestin 2, receptor desensitization and internalization may occur [14]. Endogenous and exogenous ligands may produce different effects, including respiratory depression, euphoria, and hormone release. µ- and δ-opioid receptor agonists are the predominant mediators of analgesia, whereas κ-opioid agonists are mainly associated with dysphoria. Oxycodone is a μ-opioid receptor-preferring agonist which, at higher doses, can stimulate κ-opioid receptors [15]. Evidence suggests that the antinociceptive effects of oxycodone may be partially mediated by κ-opioid receptor activation, whereas morphine mainly interacts with μ-opioid receptor subtypes [16]. Fentanyl is a potent opioid agonist widely used for severe pain that selectively binds µ-opioid receptors, while exhibiting a very low affinity for δ- and κ-opioid receptor subtypes [2].

#### 3.1.2. Metabolism

Most opioids undergo metabolism in the liver by CYP450 enzymes and, to a lesser extent, by UDP-glucuronosyltransferases (UGTs). Depending on the opioid, metabolic pathways may result in either pharmacological activation through the formation of active metabolites endowed with analgesic properties, or metabolic inactivation through the generation of inactive metabolites, with distinct implications for therapeutic efficacy and safety (Table 1). The most important isoenzymes involved in opioid metabolism are cytochrome P450 2D6 (CYP2D6) and cytochrome P450 3A4 (CYP3A4) [17]. Age, genetic polymorphisms, and pathophysiological changes, including renal and hepatic impairment, may also influence opioid metabolism [18].

Tramadol is a synthetic opioid mainly metabolized by CYP2D6 in O-desmethyl-tramadol (M1), the main pharmacologically active metabolite, which displays a higher affinity for μ-opioid receptors than the parent compound [19]. Additional metabolites are generated through CYP3A4-mediated pathways, including N-desmethyltramadol (M2), which can be further converted to O,N-didesmethyltramadol (M5), a metabolite with limited opioid activity. Overall, the formation of active metabolites, particularly M1, contributes to the analgesic efficacy and duration of tramadol action [20].

Codeine undergoes metabolic conversion to morphine, a pharmacologically active metabolite, primarily via CYP2D6. It is also metabolized to norcodeine via CYP3A4, a metabolite with weak or negligible analgesic activity, and to codeine-6-glucuronide (C-6-G), an active metabolite, by UDP-glucuronosyltransferase 2B7 (UGT2B7) [21]. UGT2B7 is also responsible for the metabolism of morphine into morphine-6-glucuronide (M-6-G), an active metabolite with analgesic properties, and morphine-3-glucuronide (M-3-G), which is considered pharmacologically inactive.

Oxycodone is a widely used opioid analgesic for the treatment of moderate to severe pain. It is primarily metabolized by CYP3A4 to noroxycodone, a major metabolite with weak affinity for μ-opioid receptors and limited analgesic activity. In parallel, CYP2D6 contributes to the formation of oxymorphone, a minor but pharmacologically active metabolite with high μ-opioid receptor affinity [22].

Buprenorphine is extensively metabolized by CYP3A4 to norbuprenorphine, a pharmacologically active metabolite, exhibiting markedly lower analgesic activity compared with the parent drug [23].

Unlike several other opioids, methadone and fentanyl do not undergo metabolic activation and therefore do not generate clinically relevant active metabolites. Fentanyl is mainly converted to the non-toxic and inactive metabolite, norfentanyl, by CYP3A4 [19]. The CYP3A4-derived inactive metabolites of methadone are 2-ethylidene-1,5-dimethyl-3,3-diphenyl-pyrrolidine (EDDP) and 2-ethyl-5-methyl-3,3-diphenyl-pyrroline (EMDP) [20].

Hydromorphone, a semi-synthetic morphine analog, undergoes extensive hepatic glucuronidation primarily via UGT2B7, resulting predominantly in hydromorphone-3-glucuronide (H-3-G), which is considered pharmacologically inactive. Minor formation of hydromorphone-6-glucuronide (H-6-G) has also been reported. These metabolic pathways occur with minimal involvement of the CYP450 system [5].

In addition, hydromorphone has been described as a minor metabolite of morphine in experimental settings [24]. Although the exact metabolic pathway has not been fully elucidated, it has been hypothesized that morphine may be oxidized to morphinone by morphine dehydrogenase and subsequently reduced to hydromorphone by morphinone reductase [24] (Table 1).

#### 3.1.3. Efflux Transporters

ATP-binding cassette sub-family B member 1 (ABCB1), also known as P-glycoprotein (P-gp) is a member of adenosine triphosphate–binding cassette (ABC) transporter superfamily, capable of binding many different substrates, including opioids [25]. Opioids can influence P-gp activity. Accordingly, morphine and oxycodone are P-gp inducers, while buprenorphine and methadone function as inhibitors of ABC transporters at the blood–brain barrier (BBB) level [26].

For example, P-gp limits the penetration of several opioids into the central nervous system (CNS) by mediating efflux across the BBB [27]. In particular, fentanyl is considered a P-gp substrate, and interindividual variability in P-gp expression or activity may contribute to differences in fentanyl central exposure and clinical response. Genetic polymorphisms in the *ABCB1* gene encoding P-gp have been correlated with reduced transporter function, resulting in an increased risk of CNS-related adverse effects of fentanyl [28], such as sedation and respiratory depression [29,30].

P-gp may also be involved in the development of central opioid tolerance by modulating brain opioid concentrations.

## 4. Adverse Drug Reactions

Opioid use is frequently associated with the development of AEs that can limit treatment effectiveness. Constipation represents one of the most common gastrointestinal AEs, with a reported prevalence ranging from approximately 40% to 95% among patients. Given its clinical relevance, prophylactic strategies aimed at maintaining adequate bowel function are commonly recommended in patients receiving opioid therapy. These include non-pharmacological measures, such as adequate hydration, increased dietary fiber intake and physical activity. When these approaches are ineffective, pharmacological interventions including stool softeners (e.g., sorbitol), stimulant laxatives such as senna, or other laxatives may be employed. Among the different opioids, morphine appears to be the most strongly associated with constipation. Although evidence regarding the route of administration is scarce, transdermal fentanyl may represent a valid alternative in patients suffering from severe opioid-induced constipation.

In addition, nausea and vomiting are the other two most common gastrointestinal AEs. Nausea, which occurs in approximately 25% of patients, is often transient and pharmacologically manageable. The most serious AE, although relatively uncommon but potentially life-threatening, is respiratory depression, typically associated with opioid overdose. The pharmacological treatment for opioid-induced respiratory depression is naloxone [31,32]. Long-term opioid use may also be associated with tolerance and opioid-induced hyperalgesia. Increasing daily doses may temporarily overcome tolerance but, at the same time, increase the risk of dependence and addiction [33]. Research efforts have therefore focused on the development of novel opioid compounds (e.g., tapentadol), aimed at reducing the risk of addiction while preserving the analgesic efficacy [34,35]. Moreover, progressive dose escalation may lead to opioid-induced hyperalgesia, a state of nociceptive sensitization that often requires dose tapering or treatment discontinuation [33]. Consequently, the clinical management of opioid therapy remains challenging, particularly in identifying patients at increased risk of opioid use disorder [36].

In this context, there is a growing need for novel tools, such as pharmacogenetics, capable of improving patient risk stratification and contributing to the prediction of treatment efficacy and adverse outcomes.

## 5. Pharmacogenetics

### 5.1. CYP2D6

*CYP2D6* is involved in the metabolism of several opioids. *CYP2D6* gene is highly polymorphic, with more than 100 single-nucleotide polymorphisms (SNPs) identified as key contributors to substantial interindividual variability across different ethnic and racial populations [37]. Moreover, multiple copies of the *CYP2D6* gene may be present on the same chromosome, resulting in an ultra-rapid metabolizer (UM) phenotype as depicted below [38,39]. The *CYP2D6* *1, *2, and *35 polymorphisms are associated with normal enzyme activity, whereas others may result in non-functional alleles (*CYP2D6* *3, *4, and *6) or reduced-functional alleles (*CYP2D6* *9, *10, *17, *29, and *41). In particular, the *CYP2D6* *5 allele results from complete gene deletion and confers no enzymatic activity. Based on allelic combination, four metabolizer phenotypes can be identified: poor (PM), intermediate (IM), extensive/normal (EM), and UM metabolizers [36,40]. *CYP2D6* PMs are more common in European and Jewish populations, IMs in African and African American populations, and EMs in East Asians and South-Central Asians. *CYP2D6* UMs are more frequently observed in Jewish and Middle Eastern population than in other ethnic groups [36]. Reduced or absent CYP2D6 activity may result in limited or absent conversion of opioid prodrugs into their active metabolites, potentially requiring dose adjustment to maintain therapeutic efficacy. Conversely, UMs generate higher levels of active metabolites, which is associated with an increased risk of AEs (Table 2) [41,42].

### 5.2. CYP3A4/5

Most of the genetic polymorphisms found in the *CYP3A4* gene result in a reduced enzyme activity, with *CYP3A4* *1B, *2, *3, and *22, being the most relevant in terms of phenotypic change [43]. Several lines of evidence have demonstrated that heterozygous patients carrying the *CYP3A4* *22 allele exhibit a 47% reduction in fentanyl clearance [43]. CYP3A5 metabolizes many of the same substrates as CYP3A4 and marked interethnic variability in CYP3A5 expression has been reported, largely attributable to common genetic variants, including the *CYP3A5* *1, *3, *6, and *7 alleles [44,45]. The *3, *6, and *7 alleles result in the production of non-functional truncated proteins, whereas the *1 allele is associated with normal enzymatic activity [46,47]. In approximately 70% of Caucasian individuals, the *3 variant is expressed resulting in absent enzyme activity [48]. In Takashina et al.’s study, conducted in cancer patients switched to transdermal fentanyl, the *CYP3A5* *3 variant was associated with higher plasma fentanyl concentration and a greater incidence of CNS AEs than the *1 variant (Table 2) [30].

### 5.3. CYP2B6

CYP2B6 exhibits substantial genetic polymorphisms and is mainly involved in methadone metabolism. The 516G>T and 785A>G polymorphisms defining the *CYP2B6* *6 allele have been associated with reduced enzymatic activity, and individuals homozygous for these variants may require lower methadone doses than heterozygous non-carrier subjects [49,50]. However, studies investigating the impact of CYP2B6 genotype on methadone PK have yielded conflicting results (Table 2) [51,52].

### 5.4. UGT2B7

Another enzyme involved in opioid metabolism is UGT2B7, which catalyzes the conversion of morphine into M-3-G and M-6-G, the latter being the active metabolite responsible for the analgesic effects. The most extensively studied *UGT2B7* polymorphisms are 802 T>C (rs7439366) and 900G>A (rs7438135). The distribution of these alleles shows significant ethnic variability; for example, the 802CC genotype is highly prevalent in Caucasian populations (~50%) but less frequent in East Asian cohorts [52,53]. Patients carrying the UGT2B7 802CC genotype appear to exhibit increased morphine metabolism and a higher M-6-G/M-3-G ratio compared with T-allele carriers, potentially requiring lower morphine doses. In contrast, some studies have reported that individuals carrying the 802T allele experience greater analgesia, likely due to reduced glucuronidation activity, whereas other investigations have found no significant association between this variant and clinical response. Overall, the clinical relevance of the 802T>C polymorphism remains controversial [53,54].

Similarly, although some reports suggest that the *UGT2B7* 900G>A variant is associated with higher enzymatic activity than the wild-type *UGT2B7* 900G, other studies have reported no significant impact on morphine PK [53,54,55]. Despite these inconsistencies, no statistically significant differences in the plasma concentration of morphine and its metabolites were observed between the different genotypes. However, multivariate stepwise linear regression analyses have occasionally identified a modest but statistically significant association between the 802CC genotype and morphine dose requirements (Table 2) [52,56].

### 5.5. P-gp

Opioid PK can also be influenced by the activity of membrane transporters, such as P-gp (P-glycoprotein), encoded by the *ABCB1* gene, which actively transports drugs out of CNS. Among the 50 SNPs identified, those of greatest interest are c.1236C>T, c.2677G>T/A, and c.3435C>T, located in exons 12, 21 and 26, respectively. These polymorphisms are more frequent in Caucasian and Asian populations than in African populations [52,53,54,55,57]. A study in healthy individuals carrying the c.3435TT genotype showed a reduced transporter expression at the duodenal level [58], which could potentially influence P-gp expression at the BBB. This finding correlates with the higher cerebrospinal fluid concentrations of morphine observed after intravenous infusion in c.3435TT subjects. Consequently, patients harboring this genetic variant have a higher risk of opioid-related AEs, potentially requiring dose reduction [59]. Rhodin et al. conducted a study in patients with chronic back pain treated with remifentanil and reported an increased frequency of AEs, including sweating, sedation, tension, and stress in homozygous c.3435T/T carriers compared with heterozygous c.3435C/T and homozygous c.3435C/C [60]. Moreover, another study investigated the association between c.3435C/T SNP in the *ABCB1* gene and opioid consumption for post-operative pain control in 152 patients undergoing nephrectomy. The observed involvement of *ABCB1* polymorphisms in opioid consumption supports their potential role in guiding acute postoperative pain management [61]. Regarding the c.1236C>T polymorphism, Fujita et al. observed a higher frequency of fatigue after morphine administration in CC individuals compared with TT carriers. This finding was further supported by higher morphine clearance in c.1236TT individuals [62]. Another study demonstrated that the c.2677G>T/A and the c.1236C>T SNPs in the *ABCB1* gene were associated with a lower incidence of CNS AEs, such as drowsiness, confusion, and hallucinations, after morphine administration (Table 2) [63]. Methadone is also a substrate of P-gp. In in vivo rat studies, P-gp inhibitors resulted in higher brain methadone concentrations and a more pronounced analgesic effect [64]. The 1236T, 2677T and 3435T variants of the *ABCB1* gene have been shown to reduce P-gp activity in vitro. In addition, individuals carrying the homozygous polymorphic haplotype (i.e., TT-TT-TT at loci rs1045642, rs2032582, and rs1128503) exhibited an approximately five-fold increased probability of requiring higher methadone doses. In contrast, heterozygous carriers for these three SNPs were about three-fold more likely to achieve adequate analgesia at lower methadone doses [65].

### 5.6. Opioid Receptors (OPRs)

The opioid receptor mu 1 (*OPRM1*) gene encodes the µ-opioid receptor. The c.118A>G polymorphism results in an amino acids substitution in the extracellular domain of the receptor, leading to reduced opioid binding affinity. The 118G allele is more frequent in Asian populations (40–50%), shows intermediate prevalence in European populations (15–30%), and is infrequent in African populations [49]. Clinical studies in postoperative pain patients treated with morphine or fentanyl have shown that individuals carrying the polymorphic G allele require higher opioid doses than wild-type A/A homozygotes [66]. Another study reported that *OPRM1* c.118A/A homozygotes experienced greater pain relief following opioid treatment than G/G homozygotes, whereas no significant difference was observed between A/G heterozygotes and G/G homozygotes. These findings suggest that individuals carrying at least one G allele may exhibit reduced responsiveness to morphine compared with A/A homozygotes [53,67]. The *OPRM1* 118G allele has been associated with several phenotypic traits, including variability in opioid response and susceptibility to opioid dependence, although results across studies remain heterogeneous. In addition, comorbid substance use disorders, such as alcohol use disorder, have been reported to alter hypothalamic–pituitary–adrenal axis reactivity to stress, potentially confounding opioid responsiveness in clinical settings. Furthermore, several studies have reported a positive association between the 118G allele and response to naltrexone, an opioid antagonist [48,68,69]. The κ-opioid receptor (KOR) is encoded by the opioid receptor kappa 1 (*OPRK1*) gene. KOR plays a role in pain perception and mediates the hypolocomotor effects, analgesia, and AEs of synthetic opioids. Genetic variations of OPRK1 have been associated with alcohol dependence and opioid addiction. Moreover, *OPRK1* genetic variability has been shown to modulate methadone efficacy, with the rs3802279 CC, rs3802281 TT, and rs963549 CC genotypes being associated with lower daily methadone maintenance dose. Notably, the rs10958350-rs7016778-rs12675595 haplotype has been associated with opioid withdrawal symptoms [7,70].

### 5.7. COMT

The catechol-O-methyltransferase (COMT) gene encodes the COMT enzyme, which plays a key role in the metabolism of catecholamines and contributes to the modulation of pain perception. Through its effects on central neurotransmitter signaling, COMT activity may indirectly influence μ-opioid receptor–mediated pathways involved in analgesic response [71,72,73]. In subjects carrying the c.472G>A genotype, lower concentrations of metenkephalin and higher MOR expression have been reported [73]. In agreement with these findings, these individuals have been shown to require lower opioids doses for neoplastic and postoperative pain management [74,75,76,77,78,79]. Several SNPs in the *COMT* gene, including c.1-98A>G (rs62699), 186C>T (rs4633), c.408C>G (rs4818), and c.472G>A (rs4680), have been associated with differences in opioid response. In the study by Lotta et al., the three genotypes associated with the c.472G>A variant were linked to distinct levels of COMT enzymatic activity. Accordingly, individuals with A/A homozygous genotype exhibit lower enzyme activity and higher pain sensitivity, G/A heterozygotes show intermediate activity, whereas G/G homozygotes display higher enzyme activity and lower pain sensitivity [80]. These observations were further supported by Henker et al., who reported that G/G homozygous and A/G heterozygous patients experienced lower postoperative pain scores compared with A/A homozygous individuals during opioid treatment [81]. In contrast, another study focusing on morphine use in neoplastic pain management reported a need for higher opioid doses in individuals with the G/G genotype compared with A/G and A/A genotypes. The authors suggested differences in opioid dose requirements may reflect COMT-related modulation of central pain processing pathways, thereby influencing opioid responsiveness compared with G/G individuals [82].

### 5.8. OCT1

Organic cation transporter 1 (OCT1) is an influx transporter encoded by the solute carrier family 22-member 1 (*SLC22A1*) gene, predominantly expressed in the liver and recognizes morphine and tramadol as substrates. The *SLC22A1* gene is highly polymorphic; compared to the wild-type gene allele, the variants *SLC22A1* *2, *3, *4, *5, and *6, are associated with loss of OCT1 activity, resulting in reduced hepatic uptake and increased plasma concentration of morphine and tramadol, thereby potentially altering treatment efficacy. Furthermore, carriers of only one active *SLC22A1* allele and carriers of no active *SLC22A1* alleles exhibit a 1.5-fold and 1.7-fold increase, respectively, in the area under the plasma morphine concentration–time curve compared with carriers of two active alleles. In contrast, the study by Tzvetkov et al. reported that plasma tramadol concentrations were not significantly affected by *SLC22A1* genotypes; however, the disposition of M1 appeared to be influenced by OCT1 transporter activity [78]. Specifically, OCT1 overexpression increased M1 uptake by approximately 2.4-fold, whereas this effect was not observed when loss-of-function *SLC22A1* variants were expressed. Finally, children homozygous for loss-of-functions *SLC22A1* variants have been shown to exhibit significantly reduced opioid clearance, further supporting the role of OCT1 genetic variability in modulating opioid PK [53,83,84].

### 5.9. ARRB2 and DCC

A recent prospective, multicenter, open-label study investigated the correlation between polymorphisms in the *β2-arrestin* (*ARRB2*) gene and clinical response to methadone administered to neoplastic pain in advanced-stage cancer patients [85,86]. In this context, significant associations were identified between pain scores and *ARRB2* variants rs3786047, rs1045280 (c.840C>T), and rs2036657. The results suggested that polymorphisms in *ARRB2* gene influence response to methadone and pain relief [87]. Finally, heterozygous variants of the Netrin 1 Receptor gene (*DCC*) have been associated with a reduced propensity to develop opioid-induced hyperalgesia following chronic morphine administration [88]. Taken together, these studies highlight the pivotal role of opioids pharmacogenetics in guiding opioid selection and optimizing pain management, particularly in the context of personalized medicine [89]. For these reasons, further randomized controlled trials are critically needed to clarify the clinical relevance of these biomarkers across different clinical settings and to support their translation into routine clinical practice.

## 6. Opioids Interactions

### 6.1. Drug–Drug Interactions

Co-administration of drugs capable of inducing or inhibiting enzymes involved in opioid metabolism may generate clinically relevant drug–drug interactions. Induction of CYP450 isoenzymes that metabolize opioid prodrugs can lead to reduced analgesic efficacy, as reported for oxycodone [90,91]. Conversely, inhibition of CYP3A4 and CYP2D6 may increase the risk of opioid-induced toxicity, since these enzymes catalyze the conversion of active parent compounds into inactive metabolites, as observed with methadone and fentanyl [90,92].

Antidepressant drugs, including fluoxetine and paroxetine [93], as well as bupropion [94], inhibit CYP2D6, thereby increasing plasma concentrations of parent tramadol. This PK interaction reduces the formation of the active metabolite M1, particularly its dextrorotatory (+)-enantiomer, which exhibits high MOR affinity and accounts for most of tramadol’s opioid-mediated analgesic effect. As a result, CYP2D6 inhibition may reduce analgesic efficacy while increasing exposure to unmetabolized tramadol. The clinical relevance of this interaction is further explained by the enantiomer-specific pharmacology of tramadol. While analgesia is largely mediated by M1 through MOR activation, the parent compound exerts additional monoaminergic effects. Specifically, the levorotatory (–) enantiomer predominantly inhibits norepinephrine reuptake, whereas the dextrorotatory (+) enantiomer inhibits serotonin reuptake and displays modest MOR binding. Increased exposure to the (+)-tramadol enantiomer enhances synaptic serotonin levels, potentially resulting in excessive activation of central 5-HT receptors, particularly 5-HT1A and 5-HT2A, thereby providing a mechanistic basis for serotonergic AEs, including serotonin syndrome, especially in the presence of other serotonergic agents or impaired metabolic conversion to M1.

In addition, the CYP2D6-mediated conversion of codeine to morphine may be impaired by the concomitant use of selective serotonin reuptake inhibitors resulting in a loss of analgesic efficacy [95], even though atypical opioids (e.g., tramadol and tapentadol) also act through the inhibition of serotoninergic and noradrenergic descendent pathways able to modulate nociception at the spinal level. The same mechanism may explain the reduced analgesic efficacy observed in patients receiving tramadol or codeine [84,92] concomitantly with cimetidine, an H2-receptor antagonist. Furthermore, antiarrhythmic drugs, such as quinidine and amiodarone, may also interact with opioids. Quinidine and the active N-derivative of monodesethyl-amiodarone metabolite of amiodarone [95], have been shown to reduce CYP2D6-mediated activation of codeine and tramadol, respectively [93]. Ondansetron, an antiemetic used to control tramadol-induced nausea and vomiting, may also reduce the M1 formation through metabolic competition at CYP2D6. Therefore, it is important to assess whether adequate analgesia is achieved and to adjust tramadol dosing accordingly. In addition, concurrent treatment should be discontinued if serotonin syndrome occurs. The antiretroviral agent ritonavir is another potent CYP2D6 inhibitor that can impair the analgesic efficacy of codeine [94].

Unlike opioid prodrugs, oxycodone-induced analgesia is primarily mediated by the parent compound. Consequently, induction of CYP3A-mediated metabolism may lead to treatment failure, while enhanced opioid effects are expected when oxycodone is combined with potent CYP3A inhibitors [90]. For example, the induction of CYP2D6 and CYP3A4 by rifampicin significantly reduces oxycodone plasma concentrations after both oral and intravenous administration [92,93]. Conversely, inhibition of hepatic and/or intestinal CYP3A activity by azole antifungals results in increased oral oxycodone exposure in healthy subjects, thereby enhancing analgesic effects and increasing the risk of serious AEs [78,79]. Methadone and fentanyl are potent analgesic opioids primarily metabolized by CYP3A4 [90,92]. CYP3A4 inhibition by antiretroviral or antimicrobial agents leads to elevated fentanyl plasma concentrations, with associated risk of respiratory depression (Table 3) [90]. Similar clinical consequences have been observed in patients receiving methadone in combination with ritonavir, ketoconazole, itraconazole, ciprofloxacin, clarithromycin, and the Ca^2+^ antagonist, diltiazem (Table 3) [92]. Table 3 summarizes clinically relevant drug–drug interactions affecting the metabolism of commonly used opioids through CYP3A4 and CYP2D6 induction or inhibition, highlighting their impact on therapeutic efficacy and AEs risk.

In addition to respiratory depression, concomitant use of methadone with drugs that inhibit its metabolism may increase the incidence of QTc interval prolongation and torsades de pointes [94,95]. Conversely, induction of CYP3A4 activity by antibiotics, anticonvulsant/antiepileptic drugs, and barbiturates can lead to the development of opioid withdrawal syndrome symptoms [95]. To date, limited evidence is available regarding drugs capable of modulating UGT activity. Some in vivo studies have reported alterations in morphine metabolite profiles, specifically M-3-G and M-6-G, following co-administration with other pharmacological agents. However, further investigations are required to clarify the extent to which the different drugs contribute to modulation of UGT activity and its clinical relevance, which remains incompletely understood [90].

### 6.2. Herb-Food Interactions

CYP3A4 and CYP2D6 can also be inhibited or induced by some herbs and food [19]. Strong or moderate CYP3A4 inhibitors also include specific foods and herbal products, such as Seville orange, lime, cranberry, and goldenseal, whereas ginseng and licorice have been reported to act as CYP3A4 inducers. Ginkgo biloba and piperine- or pepper-derived extracts represent notable exceptions, as they may exert dual modulatory effects on CYP3A4 activity. However, the most well-characterized interactions between opioids and herbal or dietary products involve grapefruit juice, a CYP3A4 inhibitor, and Saint John’s wort, a CYP3A4 inducer. Overall, the likelihood of clinically relevant interactions between herbal products or foods and CYP2D6 substrates appears to be lower than that observed for CYP3A4 substrates. Among herbal products, goldenseal and black seed have been identified as strong CYP2D6 inhibitors, whereas ginseng and kudzu act as mild to moderate inhibitors; notably, no clinically relevant CYP2D6 inducers have been identified to date [96]. In addition, the probability and clinical significance of herb–drug or food–drug interactions may depend on several factors, including the concentration of active constituents, dose, and amount consumed.

## 7. Conclusions and Future Directions

Overall, opioid response is driven by a dynamic interplay between (i) genetic variability in key metabolic pathways (most notably CYP2D6), (ii) concomitant medications that inhibit or induce these pathways and may shift the functional phenotype (including inhibitor-mediated phenoconversion), and (iii) patient- and indication-specific factors. From a clinical perspective, structured medication reconciliation, and systematic drug–drug interaction screening represents key considerations when initiating opioid therapy or adjusting doses, particularly in the context of CYP2D6- or CYP3A4-modulating co-medications.

Among currently available pharmacogenetic tools, the most actionable evidence supports CYP2D6-guided approaches for activation-dependent opioids, such as codeine and tramadol, whereas evidence for other opioids and candidate biomarkers (e.g., CYP2D6 for oxycodone or methadone; OPRM1 and COMT variants) remains heterogeneous and insufficient to support broad routine implementation [38]. Future research, including PRISMA-guided systematic reviews and well-designed clinical studies, should prioritize clinically meaningful endpoints (e.g., analgesic efficacy, AEs, dose requirements), include diverse populations, and explicitly model the combined effects of genotype and interacting co-medications. In this context, electronic clinical decision support systems integrating pharmacogenetic interpretation with real-time drug–drug interaction alerts may represent a valuable framework for advancing individualized opioid therapy.

## Figures and Tables

**Table 1:** Overview of active and inactive metabolites produced through CYP450 and UGT2B7 enzymatic metabolism of the commonly prescribed opioid drugs.

	CYP3A4		CYP2D6		UGT2B7	
	Active	Inactive	Active	Inactive	Active	Inactive
Codeine	NORC ^§^		Morphine		C-6-G	
Morphine					M-6-G	M-3-G
Tramadol		M2	M1, M5 ^§^			
Fentanyl		Norfentanyl				
Hydromorphone *						H-6-G, H-3-G
Buprenorphine	Norbuprenorphine					
Oxycodone	Noroxycodone ^§^		Oxymorphone			
Methadone		EDDP, EMDP				

* Hydromorphone is in turn an active metabolite of morphine; ^§^ Metabolites with weak affinity for opioid receptors and limited analgesic activity. Abbreviations: NORC: Norcodeine; C-6-G: Codeine-6-glucuronide; M-6-G: Morphine-6-glucuronide; M-3-G: Morphine-3-glucuronide; M2: N-desmethyl-tramadol; M1: O-desmethyl-tramadol; M5: O,N-didesmethyl-tramadol; H-6-G: Hydromorphone-6-glucoronide; H-3-G: Hydromorphone-3-glucuronide; EDDP: 2-ethylidene-1,5-dimethyl-3,3-diphenylpyrrolidine; EMDP: 2-ethyl-5-methyl-3,3-diphenylpyrroline; CYP2D6: Cytochrome P450 family 2 subfamily D member 6; CYP3A4: Cytochrome P450 family 3 subfamily A member 4; UGT2B7: UDP-glucuronosyltransferase family 2 subfamily B member 7.

**Table 2:** Polymorphisms in genes encoding enzymes and transporters involved in opioid metabolism and disposition, and their reported associations with commonly used opioid drugs.

Gene	Polymorphism	Drug
CYP2D6	*1, *2, *35	Tramadol
	*3, *4, *6	codeine
	*9, *10, *17, *29, *41	Hydromorphone
CYP3A4	*1b, *2, *3, *22	Codeine, oxycodone, buprenorphine, fentanyl
CYP3A5	*1b, *2, *3, *22	Methadone, fentanyl, alfentanil
CYP2B6	*6	Methadone
ABCB1	1236C>T, 3435C>T and 2677G>T/A	Morphine, fentanyl
UGT2B7	802T>C and 900G>A	Morphine

Abbreviations: CYP2D6: Cytochrome P450 family 2 subfamily D member 6; CYP3A4: Cytochrome P450 family 3 subfamily A member 4; CYP3A5: Cytochrome P450 family 3 subfamily A member 5; CYP2B6: Cytochrome P450 family 2 subfamily B member 6; ABCB1: ATP-binding cassette sub-family B member 1; UGT2B7: UDP-glucuronosyltransferase family 2 subfamily B member 7.

**Table 3:** Clinically relevant drug–drug interactions affecting the metabolism of commonly used opioid drugs through induction or inhibition of CYP3A4 and CYP2D6, and their potential impact on therapeutic efficacy and related adverse effects.

Drug	CYP3A4		CYP2D6		Clinical Consequences
	Inducer	Inhibitor	Inducer	Inhibitor	
Tramadol	NR	NR	NR	Antidepressants (fluoxetine, paroxetine, bupropion)	Reduced formation of the active metabolite (O-desmethyltramadol) and decreased analgesic efficacy
Codeine	NR	NR	NR	Antidepressants (fluoxetine, paroxetine, bupropion) Antihistamines (cimetidine) Antiarrhythmic (amiodarone, quinidine) Antiemetics (ondansetron) Antiretrovirals (ritonavir)	Reduced conversion to morphine and decreased analgesic effect
Oxycodone	Antibiotic (rifampicin)	NR	Antibiotic (rifampicin)	NR	Reduced plasma concentration and analgesia
	NR	Antifungals (voriconazole, itraconazole, ketoconazole)	NR	NR	Increased plasma concentration and risk of sedation, nausea, hypotension, respiratory depression
Fentanyl	NR	Antiretrovirals (ritonavir, nelfinavir) Antifungals (voriconazolo, ketoconazole, itraconazole) Antibiotics (ciprofloxacin, troleandomycin, clarithromycin)	NR	NR	Increased plasma levels and risk of respiratory depression and bradycardia
Methadone	Antibiotics (rifampicin) Anticonvulsants (carbamazepine) Antiepileptics (phenytoin) Barbiturates (pentobarbital)	NR	NR	NR	Decreased plasma level and withdrawal risk
	NR	Antiretroviral (ritonavir, nelfinavir) Antifungals (voriconazolo, ketoconazole, itraconazole), Antibiotics (ciprofloxacin, troleandomycin clarithromycin) Calcium Channel Blocker (diltiazem)	NR	NR	Increased plasma level and risk of sedation, confusion, respiratory depression, QT prolongation

Abbreviations: CYP2D6: Cytochrome P450 family 2 subfamily D member 6; CYP3A4: Cytochrome P450 family 3 subfamily A member 4; NR: not reported.

## Data Availability

No new data were created or analyzed in this study. Data sharing is not applicable to this article.

## References

[1] Zöllner C., Stein C. (2007). Opioids. Handb. Exp. Pharmacol..

[2] Coates S., Lazarus P. (2023). Hydrocodone, Oxycodone, and Morphine Metabolism and Drug–Drug Interactions. J. Pharmacol. Exp. Ther..

[3] Opioid Dispensing Rate Maps|Overdose Prevention|CDC. https://www.cdc.gov/overdose-prevention/data-research/facts-stats/opioid-dispensing-rate-maps.html.

[4] Dowell D., Ragan K.R., Jones C.M., Baldwin G.T., Chou R. (2022). CDC Clinical Practice Guideline for Prescribing Opioids for Pain—United States, 2022. MMWR Recomm. Rep..

[5] Trescot A.M., Datta S., Lee M., Hans H. (2008). Opioid Pharmacology. Pain Physician.

[6] Vearrier D., Grundmann O. (2021). Clinical Pharmacology, Toxicity, and Abuse Potential of Opioids. J. Clin. Pharmacol..

[7] Wong A.K., Somogyi A.A., Rubio J., Philip J. (2022). The Role of Pharmacogenomics in Opioid Prescribing. Curr. Treat. Options Oncol..

[8] Yang J., Bauer B.A., Wahner-Roedler D.L., Chon T.Y., Xiao L. (2020). The Modified WHO Analgesic Ladder: Is It Appropriate for Chronic Non-Cancer Pain?. J. Pain Res..

[9] Pasternak G.W. (2014). Opiate Pharmacology and Relief of Pain. J. Clin. Oncol..

[10] Zaveri N.T. (2016). Nociceptin Opioid Receptor (NOP) as a Therapeutic Target: Progress in Translation from Preclinical Research to Clinical Utility. J. Med. Chem..

[11] Paul B., Sribhashyam S., Majumdar S. (2023). Opioid Signaling and Design of Analgesics. Prog. Mol. Biol. Transl. Sci..

[12] Holden J.E., Jeong Y., Forrest J.M. (2005). The Endogenous Opioid System and Clinical Pain Management. AACN Clin. Issues.

[13] Kopecky E.A. (2019). Opioid Pharmacology: Developmental Effects on Opioid Metabolism. Clin. J. Pain.

[14] Kalso E. (2005). Oxycodone. J. Pain Symptom Manag..

[15] Umukoro N.N., Aruldhas B.W., Rossos R., Pawale D., Renschler J.S., Sadhasivam S. (2021). Pharmacogenomics of Oxycodone: A Narrative Literature Review. Pharmacogenomics.

[16] Volpe D.A., Tobin G.A.M.M., Mellon R.D., Katki A.G., Parker R.J., Colatsky T., Kropp T.J., Verbois S.L. (2011). Uniform Assessment and Ranking of Opioid μ Receptor Binding Constants for Selected Opioid Drugs. Regul. Toxicol. Pharmacol..

[17] Aapro M., Fogli S., Morlion B., Danesi R. (2024). Opioid Metabolism and Drug-Drug Interaction in Cancer. Oncologist.

[18] Barakat A. (2019). Revisiting Tramadol: A Multi-Modal Agent for Pain Management. CNS Drugs.

[19] Miotto K., Cho A.K., Khalil M.A., Blanco K., Sasaki J.D., Rawson R. (2017). Trends in Tramadol: Pharmacology, Metabolism, and Misuse. Anesth. Analg..

[20] Leppert W. (2011). CYP2D6 in the Metabolism of Opioids for Mild to Moderate Pain. Pharmacology.

[21] Lugo R.A., Kern S.E. (2004). The Pharmacokinetics of Oxycodone. J. Pain. Palliat. Care Pharmacother..

[22] Elkader A., Sproule B. (2005). Buprenorphine: Clinical Pharmacokinetics in the Treatment of Opioid Dependence. Clin. Pharmacokinet..

[23] Valtier S., Bebarta V.S. (2012). Excretion Profile of Hydrocodone, Hydromorphone and Norhydrocodone in Urine Following Single Dose Administration of Hydrocodone to Healthy Volunteers. J. Anal. Toxicol..

[24] Liu L., Xu M., Wang J., Hu Y., Huang Z. (2025). Research Progress of Hydromorphone in Clinical Application. Physiol. Res..

[25] Kesimci E., Engin A.B., Kanbak O., Karahalil B. (2012). Association between *ABCB1* Gene Polymorphisms and Fentanyl’s Adverse Effects in Turkish Patients Undergoing Spinal Anesthesia. Gene.

[26] Takashina Y., Naito T., Mino Y., Yagi T., Ohnishi K., Kawakami J. (2012). Impact of *CYP3A5* and *ABCB1* Gene Polymorphisms on Fentanyl Pharmacokinetics and Clinical Responses in Cancer Patients Undergoing Conversion to a Transdermal System. Drug Metab. Pharmacokinet..

[27] Kharasch E.D., Hoffer C., Whittington D., Sheffels P. (2003). Role of P-Glycoprotein in the Intestinal Absorption and Clinical Effects of Morphine. Clin. Pharmacol. Ther..

[28] Rodriguez M., Ortega I., Soengas I., Suarez E., Lukas J.C., Calvo R. (2004). Effect of P-Glycoprotein Inhibition on Methadone Analgesia and Brain Distribution in the Rat. J. Pharm. Pharmacol..

[29] Levran O., O’Hara K., Peles E., Li D., Barral S., Ray B., Borg L., Ott J., Adelson M., Kreek M.J. (2008). *ABCB1 (MDR1)* Genetic Variants Are Associated with Methadone Doses Required for Effective Treatment of Heroin Dependence. Hum. Mol. Genet..

[30] Imam M.Z., Kuo A., Ghassabian S., Smith M.T. (2018). Progress in Understanding Mechanisms of Opioid-Induced Gastrointestinal Adverse Effects and Respiratory Depression. Neuropharmacology.

[31] Raehal K.M., Schmid C.L., Groer C.E., Bohn L.M. (2011). Functional Selectivity at the μ-Opioid Receptor: Implications for Understanding Opioid Analgesia and Tolerance. Pharmacol. Rev..

[32] Nafziger A.N., Barkin R.L. (2018). Opioid Therapy in Acute and Chronic Pain. J. Clin. Pharmacol..

[33] Trescot A.M. (2014). Genetics and Implications in Perioperative Analgesia. Best. Pract. Res. Clin. Anaesthesiol..

[34] Gaedigk A., Twist G.P., Farrow E.G., Lowry J.A., Soden S.E., Miller N.A. (2017). In Vivo Characterization of *CYP2D6**12, *29 and *84 Using Dextromethorphan as a Probe Drug: A Case Report. Pharmacogenomics.

[35] Caudle K.E., Sangkuhl K., Whirl-Carrillo M., Swen J.J., Haidar C.E., Klein T.E., Gammal R.S., Relling M.V., Scott S.A., Hertz D.L. (2020). Standardizing *CYP2D6* Genotype to Phenotype Translation: Consensus Recommendations from the Clinical Pharmacogenetics Implementation Consortium and Dutch Pharmacogenetics Working Group. Clin. Transl. Sci..

[36] Gaedigk A., Sangkuhl K., Whirl-Carrillo M., Klein T., Steven Leeder J. (2017). Prediction of *CYP2D6* Phenotype from Genotype across World Populations. Genet. Med..

[37] Packiasabapathy S., Sadhasivam S. (2018). Gender, Genetics, and Analgesia: Understanding the Differences in Response to Pain Relief. J. Pain. Res..

[38] Crews K.R., Monte A.A., Huddart R., Caudle K.E., Kharasch E.D., Gaedigk A., Dunnenberger H.M., Leeder J.S., Callaghan J.T., Samer C.F. (2021). Clinical Pharmacogenetics Implementation Consortium Guideline for *CYP2D6*, *OPRM1*, and *COMT* Genotypes and Select Opioid Therapy. Clin. Pharmacol. Ther..

[39] Klein K., Zanger U.M. (2013). Pharmacogenomics of Cytochrome P450 3A4: Recent Progress Toward the “Missing Heritability” Problem. Front. Genet..

[40] Saiz-Rodríguez M., Ochoa D., Herrador C., Belmonte C., Román M., Alday E., Koller D., Zubiaur P., Mejía G., Hernández-Martínez M. (2019). Polymorphisms Associated with Fentanyl Pharmacokinetics, Pharmacodynamics and Adverse Effects. Basic Clin. Pharmacol. Toxicol..

[41] Bains R.K., Kovacevic M., Plaster C.A., Tarekegn A., Bekele E., Bradman N.N., Thomas M.G. (2013). Molecular Diversity and Population Structure at the Cytochrome P450 3A5 Gene in Africa. BMC Genet..

[42] Lee S.J., Usmani K.A., Chanas B., Ghanayem B., Xi T., Hodgson E., Mohrenweiser H.W., Goldstein J.A. (2003). Genetic Findings and Functional Studies of Human *CYP3A5* Single Nucleotide Polymorphisms in Different Ethnic Groups. Pharmacogenetics.

[43] Freiermuth C.E., Kisor D.F., Lambert J., Braun R., Frey J.A., Bachmann D.J., Bischof J.J., Lyons M.S., Pantalon M.V., Punches B.E. (2023). Genetic Variants Associated With Opioid Use Disorder. Clin. Pharmacol. Ther..

[44] Kreek M.J., Levran O., Reed B., Schlussman S.D., Zhou Y., Butelman E.R. (2012). Opiate Addiction and Cocaine Addiction: Underlying Molecular Neurobiology and Genetics. J. Clin. Investig..

[45] Sim S.C., Ingelman-Sundberg M. (2010). The Human Cytochrome P450 (CYP) Allele Nomenclature Website: A Peer-Reviewed Database of CYP Variants and Their Associated Effects. Hum. Genom..

[46] Zanger U.M., Klein K. (2013). Pharmacogenetics of Cytochrome P450 2B6 (CYP2B6): Advances on Polymorphisms, Mechanisms, and Clinical Relevance. Front. Genet..

[47] Wang S.C., Ho I.K., Tsou H.H., Tian J.N., Hsiao C.F., Chen C.H., Tan H.K.L., Lin L., Wu C.S., Su L.W. (2011). *CYP2B6* Polymorphisms Influence the Plasma Concentration and Clearance of the Methadone S-Enantiomer. J. Clin. Psychopharmacol..

[48] Ofoegbu A., Ettienne E.B. (2021). Pharmacogenomics and Morphine. J. Clin. Pharmacol..

[49] Bayrak A., Bayır A., Karabulut K.U., Ragab D., Elghawaby H., Eldesouky M., Elsayed T., Samir M., Nagi K., Refaie M. (2011). UDP Glucuronosyltransferase 2B7 Single Nucleotide Polymorphism (Rs7439366) Influences Heat Pain Response in Human Volunteers after i.v. Morphine Infusion. Crit. Care.

[50] Matic M., Norman E., Rane A., Beck O., Andersson M., Elens L., Tibboel D., Fellman V., Van Schaik R.H.N. (2014). Effect of *UGT2B7* -900G>A (-842G>A.;Rs7438135) on Morphine Glucuronidation in Preterm Newborns: Results from a Pilot Cohort. Pharmacogenomics.

[51] Bastami S., Gupta A., Zackrisson A.L., Ahlner J., Osman A., Uppugunduri S. (2014). Influence of *UGT2B7*, *OPRM1* and *ABCB1* Gene Polymorphisms on Postoperative Morphine Consumption. Basic Clin. Pharmacol. Toxicol..

[52] Fujita K.I., Ando Y., Yamamoto W., Miya T., Endo H., Sunakawa Y., Araki K., Kodama K., Nagashima F., Ichikawa W. (2010). Association of *UGT2B7* and *ABCB1* Genotypes with Morphine-Induced Adverse Drug Reactions in Japanese Patients with Cancer. Cancer Chemother. Pharmacol..

[53] Hoffmeyer S., Burk O., Von Richter O., Arnold H.P., Brockmöller J., Johne A., Cascorbi I., Gerloff T., Roots I., Eichelbaum M. (2000). Functional Polymorphisms of the Human Multidrug-Resistance Gene: Multiple Sequence Variations and Correlation of One Allele with P-Glycoprotein Expression and Activity in Vivo. Proc. Natl. Acad. Sci. USA.

[54] Meineke I., Freudenthaler S., Hofmann U., Schaeffeler E., Mikus G., Schwab M., Prange H.W., Gleiter C.H., Brockmöller J. (2002). Pharmacokinetic Modelling of Morphine, Morphine-3-Glucuronide and Morphine-6-Glucuronide in Plasma and Cerebrospinal Fluid of Neurosurgical Patients after Short-Term Infusion of Morphine. Br. J. Clin. Pharmacol..

[55] Rhodin A., Grönbladh A., Ginya H., Nilsson K.W., Rosenblad A., Zhou Q., Enlund M., Hallberg M., Gordh T., Nyberg F. (2013). Combined Analysis of Circulating β-Endorphin with Gene Polymorphisms in *OPRM1*, *CACNAD2* and *ABCB1* Reveals Correlation with Pain, Opioid Sensitivity and Opioid-Related Side Effects. Mol. Brain.

[56] Candiotti K., Yang Z., Xue L., Zhang Y., Rodriguez Y., Wang L., Hao S., Gitlin M. (2013). Single-Nucleotide Polymorphism C3435T in the *ABCB1* Gene Is Associated with Opioid Consumption in Postoperative Pain. Pain Med..

[57] Martin da Silva I., Plaza-Díaz A., Ruiz-Ramos J., Juanes-Borrego A., Riera P. (2025). The Role of Pharmacogenetic Biomarkers in Pain. Biomedicines.

[58] Nielsen L.M., Olesen A.E., Branford R., Christrup L.L., Sato H., Drewes A.M. (2015). Association Between Human Pain-Related Genotypes and Variability in Opioid Analgesia: An Updated Review. Pain. Pract..

[59] Campa D., Gioia A., Tomei A., Poli P., Barale R. (2008). Association of *ABCB1/MDR1* and *OPRM1* Gene Polymorphisms with Morphine Pain Relief. Clin. Pharmacol. Ther..

[60] Sturgess J.E., George T.P., Kennedy J.L., Heinz A., Müller D.J. (2011). Pharmacogenetics of Alcohol, Nicotine and Drug Addiction Treatments. Addict. Biol..

[61] Deb I., Chakraborty J., Gangopadhyay P.K., Choudhury S.R., Das S. (2010). Single-Nucleotide Polymorphism (A118G) in Exon 1 of *OPRM1* Gene Causes Alteration in Downstream Signaling by Mu-Opioid Receptor and May Contribute to the Genetic Risk for Addiction. J. Neurochem..

[62] Zhang Q., Shi M., Tang H., Zhong H., Lu X. (2020). κ Opioid Receptor 1 Single Nucleotide Polymorphisms Were Associated with the Methadone Dosage. Genet. Test. Mol. Biomark..

[63] Wang S.C., Tsou H.H., Chung R.H., Chang Y.S., Fang C.P., Chen C.H., Ho I.K., Kuo H.W., Liu S.C., Shih Y.H. (2014). The Association of Genetic Polymorphisms in the κ-Opioid Receptor 1 Gene with Body Weight, Alcohol Use, and Withdrawal Symptoms in Patients with Methadone Maintenance. J. Clin. Psychopharmacol..

[64] Mercadante S., Arcuri E., Santoni A. (2019). Opioid-Induced Tolerance and Hyperalgesia. CNS Drugs.

[65] Phillips J.K., Ford M.A., Bonnie R.J. (2017). Pain Management and the Opioid Epidemic: Balancing Societal and Individual Benefits and Risks of Prescription Opioid Use.

[66] Nascimento T.D., Yang N., Salman D., Jassar H., Kaciroti N., Bellile E., Danciu T., Koeppe R., Stohler C., Zubieta J.K. (2019). Μ-Opioid Activity in Chronic TMD Pain Is Associated with *COMT* Polymorphism. J. Dent. Res..

[67] Meloto C.B., Segall S.K., Smith S., Parisien M., Shabalina S.A., Rizzatti-Barbosa C.M., Gauthier J., Tsao D., Convertino M., Piltonen M.H. (2015). *COMT* Gene Locus: New Functional Variants. Pain.

[68] Kowarik M.C., Einhäuser J., Jochim B., Büttner A., Tölle T.R., Riemenschneider M., Platzer S., Berthele A. (2012). Impact of the *COMT* Val 108/158Met Polymorphism on the Mu-Opioid Receptor System in the Human Brain: Mu-Opioid Receptor, Met-Enkephalin and Beta-Endorphin Expression. Neurosci. Lett..

[69] Matsuoka H., Arao T., Makimura C., Takeda M., Kiyota H., Tsurutani J., Fujita Y., Matsumoto K., Kimura H., Otsuka M. (2012). Expression Changes in Arrestin β 1 and Genetic Variation in Catechol-O-Methyltransferase Are Biomarkers for the Response to Morphine Treatment in Cancer Patients. Oncol. Rep..

[70] Rakvåg T.T., Ross J.R., Sato H., Skorpen F., Kaasa S., Klepstad P. (2008). Genetic Variation in the Catechol-O-Methyltransferase (*COMT*) Gene and Morphine Requirements in Cancer Patients with Pain. Mol. Pain.

[71] De Gregori M., Garbin G., De Gregori S., Minella C.E., Bugada D., Lisa A., Govoni S., Regazzi M., Allegri M., Ranzani G.N. (2013). Genetic Variability at *COMT* but Not at *OPRM1* and *UGT2B7* Loci Modulates Morphine Analgesic Response in Acute Postoperative Pain. Eur. J. Clin. Pharmacol..

[72] Kolesnikov Y., Gabovits B., Levin A., Voiko E., Veske A. (2011). Combined Catechol-O-Methyltransferase and Mu-Opioid Receptor Gene Polymorphisms Affect Morphine Postoperative Analgesia and Central Side Effects. Anesth. Analg..

[73] Henker R.A., Lewis A., Dai F., Lariviere W.R., Meng L., Gruen G.S., Sereika S.M., Pape H., Tarkin I.S., Gowda I. (2013). The Associations between *OPRM 1* and *COMT* Genotypes and Postoperative Pain, Opioid Use, and Opioid-Induced Sedation. Biol. Res. Nurs..

[74] Xu S., Wu N., Liu X., Zhu J., Liu Z. (2024). The Catechol-O-Methyltransferase (*COMT*) Val158Met Polymorphism Is Associated with Oxycodone Requirements, Adverse Effects, and Pain Sensitivity in Cancer Patients. J. Clin. Pharm. Ther..

[75] Matsuoka H., Makimura C., Koyama A., Fujita Y., Tsurutani J., Sakai K., Sakamoto R., Nishio K., Nakagawa K. (2017). Prospective Replication Study Implicates the Catechol-O-Methyltransferase Val158Met Polymorphism as a Biomarker for the Response to Morphine in Patients with Cancer. Biomed. Rep..

[76] Goswami S., Gong L., Giacomini K., Altman R.B., Klein T.E. (2014). PharmGKB Summary: Very Important Pharmacogene Information for *SLC22A1*. Pharmacogenet. Genom..

[77] Tzvetkov M.V., Dos Santos Pereira J.N., Meineke I., Saadatmand A.R., Stingl J.C., Brockmöller J. (2013). Morphine Is a Substrate of the Organic Cation Transporter OCT1 and Polymorphisms in *OCT1* Gene Affect Morphine Pharmacokinetics after Codeine Administration. Biochem. Pharmacol..

[78] Tzvetkov M.V., Saadatmand A.R., Lötsch J., Tegeder I., Stingl J.C., Brockmöller J. (2011). Genetically Polymorphic *OCT1*: Another Piece in the Puzzle of the Variable Pharmacokinetics and Pharmacodynamics of the Opioidergic Drug Tramadol. Clin. Pharmacol. Ther..

[79] Ozberk D., Haywood A., Sutherland H.G., Yu C., Albury C.L., Zunk M., George R., Good P., Griffiths L.R., Hardy J. (2022). Association of Polymorphisms in *ARRB2* and Clinical Response to Methadone for Pain in Advanced Cancer. Pharmacogenomics.

[80] Donaldson R., Sun Y., Liang D.Y., Zheng M., Sahbaie P., Dill D.L., Peltz G., Buck K.J., Clark J.D. (2016). The Multiple PDZ Domain Protein Mpdz/MUPP1 Regulates Opioid Tolerance and Opioid-Induced Hyperalgesia. BMC Genom..

[81] Liang D.Y., Zheng M., Sun Y., Sahbaie P., Low S.A., Peltz G., Scherrer G., Flores C., Clark J.D. (2014). The Netrin-1 Receptor DCC Is a Regulator of Maladaptive Responses to Chronic Morphine Administration. BMC Genom..

[82] Magarbeh L., Gorbovskaya I., Le Foll B., Jhirad R., Müller D.J. (2021). Reviewing Pharmacogenetics to Advance Precision Medicine for Opioids. Biomed. Pharmacother..

[83] Lemberg K.K., Heiskanen T.E., Neuvonen M., Kontinen V.K., Neuvonen P.J., Dahl M.L., Kalso E.A. (2010). Does Co-Administration of Paroxetine Change Oxycodone Analgesia: An Interaction Study in Chronic Pain Patients. Scand. J. Pain.

[84] Armstrong S.C., Cozza K.L. (2003). Pharmacokinetic Drug Interactions of Morphine, Codeine, and Their Derivatives: Theory and Clinical Reality, Part II. Psychosomatics.

[85] Nahid N.A., Johnson J.A. (2022). CYP2D6 Pharmacogenetics and Phenoconversion in Personalized Medicine. Expert. Opin. Drug Metab. Toxicol..

[86] Klomp S.D., Manson M.L., Guchelaar H.J., Swen J.J. (2020). Phenoconversion of Cytochrome P450 Metabolism: A Systematic Review. J. Clin. Med..

[87] Cicali E.J., Elchynski A.L., Cook K.J., Houder J.T., Thomas C.D., Smith D.M., Elsey A., Johnson J.A., Cavallari L.H., Wiisanen K. (2021). How to Integrate CYP2D6 Phenoconversion Into Clinical Pharmacogenetics: A Tutorial. Clin. Pharmacol. Ther..

[88] Stamer U.M., Musshoff F., Kobilay M., Madea B., Hoeft A., Stuber F. (2007). Concentrations of Tramadol and O-Desmethyltramadol Enantiomers in Different *CYP2D6* Genotypes. Clin. Pharmacol. Ther..

[89] Ashraf M.W., Poikola S., Neuvonen M., Kiiski J.I., Kontinen V.K., Olkkola K.T., Backman J.T., Niemi M., Saari T.I. (2024). Population Pharmacokinetic Quantification of CYP2D6 Activity in Codeine Metabolism in Ambulatory Surgical Patients for Model-Informed Precision Dosing. Clin. Pharmacokinet..

[90] Armstrong S.C., Cozza K.L. (2002). Med-Psych Drug-Drug Interactions Update. Psychosomatics.

[91] McDonald M.G., Au N.T., Rettie A.E. (2015). P450-Based Drug-Drug Interactions of Amiodarone and Its Metabolites: Diversity of Inhibitory Mechanisms. Drug Metab. Dispos..

[92] Nieminen T.H., Hagelberg N.M., Saari T.I., Pertovaara A., Neuvonen M., Laine K., Neuvonen P.J., Olkkola K.T. (2009). Rifampin Greatly Reduces the Plasma Concentrations of Intravenous and Oral Oxycodone. Anesthesiology.

[93] Samer C.F., Daali Y., Wagner M., Hopfgartner G., Eap C.B., Rebsamen M.C., Rossier M.F., Hochstrasser D., Dayer P., Desmeules J.A. (2010). Genetic Polymorphisms and Drug Interactions Modulating CYP2D6 and CYP3A Activities Have a Major Effect on Oxycodone Analgesic Efficacy and Safety. Br. J. Pharmacol..

[94] Stringer J., Welsh C., Tommasello A. (2009). Methadone-Associated Q-T Interval Prolongation and Torsades de Pointes. Am. J. Health-Syst. Pharm..

[95] Lötsch J., Skarke C., Tegeder I., Geisslinger G. (2002). Drug Interactions with Patient-Controlled Analgesia. Clin. Pharmacokinet..

[96] Gougis P., Hilmi M., Geraud A., Mir O., Funck-Brentano C. (2021). Potential Cytochrome P450-Mediated Pharmacokinetic Interactions between Herbs, Food, and Dietary Supplements and Cancer Treatments. Crit. Rev. Oncol. Hematol..

